# LncRNA H19: A novel oncogene in multiple cancers

**DOI:** 10.7150/ijbs.62573

**Published:** 2021-07-25

**Authors:** Jun Yang, Manlong Qi, Xiang Fei, Xia Wang, Kefeng Wang

**Affiliations:** 1 Department of Gastroenterology, Shengjing Hospital of China Medical University, Shenyang 110004, China; 2 Department of Clinical Genetics, Shengjing Hospital of China Medical University, Shenyang 110004, China; 3 Department of Urology, Shengjing Hospital of China Medical University, Shenyang 110004, China

**Keywords:** lncRNA, H19, oncogene, cancers, metastasis

## Abstract

Long non-coding RNAs (lncRNAs) are a series of non-coding RNAs that lack open reading frameworks. Accumulating evidence suggests important roles for lncRNAs in various diseases, including cancers. Recently, lncRNA H19 (H19) became a research focus due to its ectopic expression in human malignant tumors, where it functioned as an oncogene. Subsequently, H19 was confirmed to be involved in tumorigenesis and malignant progression in many tumors and had been implicated in promoting cell growth, invasion, migration, epithelial-mesenchymal transition, metastasis, and apoptosis. H19 also sequesters some microRNAs, facilitating a multilayer molecular regulatory mechanism. In this review, we summarize the abnormal overexpression of H19 in human cancers, which suggests wide prospects for further research into the diagnosis and treatment of cancers.

## Introduction

More than 90% of human genomic DNA is transcribed into RNAs, but less than 2% of these nucleotide sequences encode proteins 1. Most of the transcribed RNAs are non-coding RNAs (ncRNAs), which lack of the capability to be translated into a protein. These ncRNAs are grouped according to length as long ncRNAs (lncRNAs; >200 nucleotides) and small ncRNAs (<200 nucleotides). LncRNAs are transcribed by RNA polymerase II and classified into enhancer lncRNAs, antisense lncRNAs, bidirectional lncRNAs, large intergenic ncRNAs, and intronic transcript lncRNAs 2-3. LncRNAs modulate gene expression at three levels: post-transcriptional, transcriptional, and epigenetic. Accumulating evidence has demonstrated that lncRNAs participate in many physiological and pathological processes, including apoptosis, cell proliferation, invasion, and carcinogenesis 4-5. Moreover, some lncRNAs have been identified to encode proteins.

LncRNA H19 (H19) was one of the first discovered lncRNAs and is encoded by the H19 gene 6. The H19 gene is located in the region of chromosome 11p15.5 and transcribed by RNA polymerase II, spliced, and polyadenylated 7. The 2.3 kb lncRNA is then exported from the nucleus to the cytoplasm. H19 is usually expressed in fetal tissues, and its expression is greatly reduced after birth. Recently, H19 was found to take part in a variety of pathological processes, such as inflammatory reactions, angiogenesis, neurogenesis, and fibrosis progression. Additionally, abnormal H19 overexpression is thought to be involved in the development and progression of cancer in many systems of human body, such as the digestive system, the respiratory system, the breast, the genitourinary system, the nervous system, and others.

The aim of this manuscript was to summarize recent findings on H19 expression in cancer and to clarify the impact of imprinting on cancer.

## H19 in various human cancers

H19 has been found to be ectopically expressed in many tumors, where it facilitates several oncogenic behaviors, such as increased cell viability, motility, growth, migration, invasion, metastasis, epithelial-mesenchymal transition (EMT), autophagy, cell cycle progression, colony formation, and glucose metabolism 8-11. H19 also exhibits anti-oncogenic properties in a small percentage of tumors, such as pituitary adenomas 12-14. Additionally, H19 performs different roles in different clinical stages of the same disease, such as thyroid carcinoma 15, 16, and retinoblastoma 17, 18. These findings imply that H19 expression might be different depending on the histological and cellular context of individual tumors.

Recently, several studies have demonstrated that H19 is involved in the clinicopathological progression of many different tumor types and is associated with clinical parameters such as tumor size, clinical stage, lymph node metastasis, distant metastasis, and overall survival (OS) 19, 20. H19 has also been found to take part in the microRNA (miRNA)-mediated network of gene regulation by influencing the activity of the downstream mRNAs that facilitate the aggressive phenotypes of these tumors. Furthermore, H19 plays a vital role in the chemotherapeutic resistance of some tumors and can be used as a potential therapeutic target 21, 22. The specific mechanisms and functional characterizations of H19 in tumors of each human system are shown in **Tables 1-8.** The mechanisms of H19 in various tumors will be further clarified below.

### The role of H19 in digestive system tumors

#### H19 in esophageal cancer (EC)

EC is a relatively rare cancer of the digestive system. Esophageal squamous cell carcinoma (ESCC) is the main pathological subtype, accounting for 90% of the global incidence of EC 23. The 5-year OS rate of early-stage ESCC is >90%, but <10% for patients with lymph node metastasis 24. However, many patients miss the opportunity for early detection because of atypical symptoms. Therefore, it is necessary to explore early diagnostic biomarkers in ESCC.

**Figure 1:**
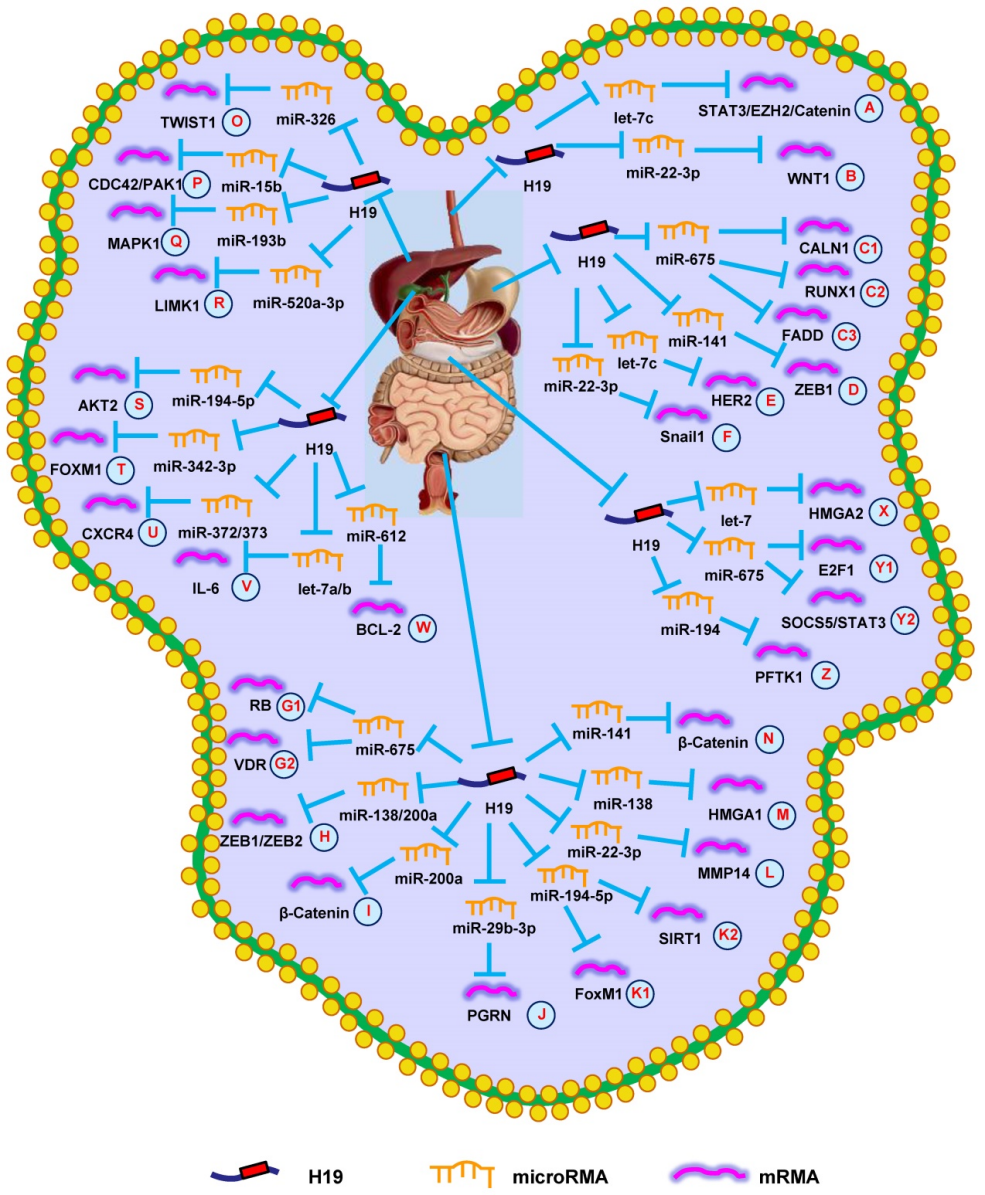
** H19 mediates mechanisms involved in digestive system tumors.** (**A**). H19 could promote the expression of STAT3/EZH2/Catenin by targeting let-7c. (**B**). H19 could promote the expression of WNT1 by targeting miR-22-3p. (**C1-C3**). H19 could promote the expression of CALN1 (**C1**), RUNX1 (**C2**), and FADD (**C3**) by targeting miR-675. (**D**). H19 could promote the expression of ZEB1 by targeting miR-141. (**E**). H19 could promote the expression of HER2 by targeting let-7c. (**F**). H19 could promote the expression of Snail1 by targeting miR-22-3p. (**G1-G2**). H19 could promote the expression of RB (**G1**) and VDR (**G2**) by targeting miR-675. (**H**). H19 could promote the expression of ZEB1/ZEB2 by targeting miR-138/200a. (**I**). H19 could promote the expression of β-Catenin by targeting miR-200a. (**J**). H19 could promote the expression of PGRN by targeting miR-29b-3p. (**K1-K2**). H19 could promote the expression of FoxM1 (**K1**) and SIRT1 (**K2**) by targeting miR-194-5p. (**L**). H19 could promote the expression of MMP14 by targeting miR-22-3p. (**M**). H19 could promote the expression of HMGA1 by targeting miR-138. (**N**). H19 could promote the expression of β-Catenin by targeting miR-141. (**O**). H19 could promote the expression of TWIST1 by targeting miR-326. (**P**). H19 could promote the expression of CDC42/PAK1 by targeting miR-15b. (**Q**). H19 could promote the expression of MAPK1 by targeting miR-193b. (**R**). H19 could promote the expression of LIMK1 by targeting miR-520a-3p. (**S**). H19 could promote the expression of AKT2 by targeting miR-194-5p. (**T**). H19 could promote the expression of FOXM1 by targeting miR-342-3p. (**U**). H19 could promote the expression of CXCR4 by targeting miR-372/373. (**V**). H19 could promote the expression of IL-6 by targeting let-7a/b. (**W**). H19 could promote the expression of BCL-2 by targeting miR-612. (**X**). H19 could promote the expression of HMGA2 by targeting let-7. (**Y1-Y2**). H19 could promote the expression of E2F-1 (**Y1**) and SOCS5/STAT3 (**Y2**) by targeting miR-675. (**Z**). H19 could promote the expression of PFTK1 by targeting miR-194.

Emerging evidence suggests that H19 is highly expressed in EC and plays an important role in EC development 25. The study by Huang et al. 26 reported that H19 expression was associated with tumor metastasis and depth in EC samples. Increased H19 expression promoted EMT, growth, and invasion of EC cells. Conversely, decreasing H19 expression inhibited the growth, migration, and invasion of ESCC cells, suggesting H19 could be a prognostic marker and therapeutic target for ESCC patients 27. Two years later, another group confirmed that downregulating H19 inhibited the growth, migration, invasion, metastasis, and EMT of EC cells by modulating the let-7c/STAT3/EZH2/β-catenin pathway (**Fig. 1A**) 28. The study by Li et al. 19 investigated the clinicopathological parameters of H19 in ESCC patients, revealing that increased H19 expression was associated with larger tumor size, poor clinical stage, and shorter OS.

Data also suggest that H19 plays a role in the efficacy of radiotherapy in ESCC patients. A team from Shandong University revealed that knocking-down H19 decreased WNT1 expression, suppressing radioresistance of ESCC cells with regards to growth and migration by upregulating miR-22-3p (**Fig. 1B**) 29. These data showed that H19 could be a prognostic marker and/or therapeutic target for EC.

#### H19 in gastric cancer (GC)

GC is an important malignant tumor of the digestive system, with China having one of the highest rates of GC in the world. Therefore, further studies of the molecular mechanisms involved in GC are urgently needed.

Nine years ago, it was first demonstrated that GC tissues showed an upregulation of H19 30. Overexpression of H19 was then found to promote cell growth *via* regulating p53 in GC. Subsequently, several groups found that H19 accelerated GC progression, demonstrating that H19 could serve as a biomarker for the early diagnosis and prognosis of GC 31-35. Zhang et al. 36 revealed that H19 promoted cell proliferation by increasing NF-κB-involved inflammation in GC. Subsequent studies showed that upregulating H19 improved the sensitivity of GC cells to X-rays and chemotherapy, leading to greater tumor weights and larger tumor sizes 21.

Recently, several research groups have provided novel insights into the active lncRNA-miRNA-mRNA network in GC. Li et al. 20 demonstrated that the effects of H19 were partially through directly upregulating ISM1 and indirectly downregulating CALN1 *via* miR-675 (**Fig. 1C1**). Similar results indicated that H19 could modulate GC progression through the miR-675/RUNX1 pathway (**Fig. 1C2**), which revealed a potential target for GC therapy 37. Just a month later, another research group came to the same conclusion 38. Additionally, the H19/miR-675 axis also participates in the development of GC through FADD/caspase 8 signaling (**Fig. 1C3**) 39. Besides miR-675, H19 has also be shown to regulate ZEB1 expression *via* sequestering miR-141, suggesting an important role of the lncRNA-miRNA functional network in GC (**Fig. 1D**) 40. The study by Wei et al. 41 indicated that H19 functioned as a competing endogenous RNA (ceRNA) to modulate HER2 expression by antagonizing let-7c in GC, providing other potential H19-based therapeutic strategies for GC (**Fig. 1E**). Subsequent studies uncovered that H19 controlled cell proliferation and metastasis through the miR-22-3p/Snail1 axis in GC (**Fig. 1F**) 42. These findings showed that H19 could provide a diagnostic option for GC.

#### H19 in colorectal cancer (CRC)

CRC is the most common tumor of the digestive system and has the third highest morbidity and mortality rate of all malignancies in the United States 43. Despite advances in early diagnosis over the last few years, the OS of CRC patients with metastases remains low. Therefore, a complete understanding of the pathogenic mechanisms of CRC represents key progress toward CRC treatments.

The study by Tsang et al. 44 revealed that H19 and its derivative, miR-675, promoted CRC cell cycle progression by targeting RB (**Fig. 1G1**). Other researchers reported that H19 accelerated cell growth and EMT by regulating ZEB1, ZEB2, and vimentin *via* functioning as a ceRNA of miR-200a and miR-138 (**Fig. 1H**) 45. Additionally, H19 could bind eIF4A3 to promote cell growth and influence tumor differentiation and TNM stage in CRC patients 46. H19 also promoted cell growth, migration, and EMT in CRC 47. Subsequent studies found that H19 promoted the proliferation, invasion, and metastasis of CRC by activating important cancer-related signaling pathways, such as RAS/MAPK 48, Rb/E2F, CDK8/β-catenin 49, and Raf/ERK 50.

Recently, several groups have focused on the lncRNA-miRNA-mRNA axis in CRC. Studies on the role of H19 in CRC revealed that H19 repressed β-catenin expression *via* binding miR-200a to promote cell growth (**Fig. 1I**) 51. Additionally, H19 was shown to be involved in EMT of CRC cells *via* the miR-29b-3p/PGRN/Wnt pathway (**Fig. 1J**) 52. Another group revealed that the H19/miR-194-5p/FoxM1 axis influenced EMT and could serve as a therapeutic target in CRC (**Fig. 1K1**) 53. Hu et al. 54 found that knocking-down HDAC2 increased H19 expression and induced EMT *via* the miR-22-3p/MMP14 pathway (**Fig. 1L**). Subsequent studies revealed that H19 increased cell migration and invasion by modulating the miR-138/HMGA1 axis, providing a novel insight for CRC treatment (**Fig. 1M**) 55.

H19 has also been proven to affect drug resistance during CRC treatment. The study by Ren et al. 56 indicated that carcinoma-associated fibroblasts increased the chemoresistance and stemness of CRC by transferring exosomal H19. Mechanistic investigations suggested that H19 can activate β-catenin signaling by functioning as a ceRNA for miR-141 (**Fig. 1N**). Another group indicated that abnormal overexpression of H19 facilitated resistance to 1,25(OH)2D3 treatment *via* the miR-675-5p/VDR axis (**Fig. 1G2**) 57. Additionally, methotrexate (MTX) resistance has impeded its application in CRC therapy. A recent study revealed that knocking-down H19 inhibited MTX resistance and promoted apoptosis *via* suppressing Wnt/β-catenin signaling in CRC 58. Subsequent studies revealed that H19 promoted 5-fluorouracil (5-Fu) resistance in CRC cells. Mechanistically, it was demonstrated that H19 led to 5-Fu resistance through miR-194-5p/SIRT1-mediated autophagy in CRC (**Fig. 1K2**) 11. These findings confirmed that H19 provided an option for suppressing CRC progression.

#### H19 in hepatocellular carcinoma (HCC)

Although the incidence of HCC is relatively low, its mortality rate is high. Because HCC frequently metastasizes, patients often have a poor prognosis. Thus, it is essential to discover effective treatments for HCC patients.

The study by Lv et al. 59 reported that aflatoxin B1 promoted E2F1 and H19 expression, which increased the growth and invasion of HCC cells. Another lab revealed that H19 expression was higher in HCC than in normal hepatic tissues, and was positively correlated with lymphatic and distant metastasis. Increasing H19 expressing promoted the progression of HCC cells by targeting miR-22 60.

Furthermore, it has been demonstrated that H19 promoted HCC progression through miRNA-mRNA pathways. Silencing H19 repressed the proliferation, migration, and invasion of HCC cells. Further investigations into the underlying mechanisms indicated that the H19/miR-326/TWIST1 axis was involved in HCC progression (**Fig. 1O**) 61. Additionally, downregulating H19 suppressed growth, migration, and invasion by regulating the miR-15b/CDC42/PAK1 axis (**Fig. 1P**) 62. Furthermore, bioinformatics analysis and *in vitro* experiments showed that H19 served as a miR-193b sponge to protect MAPK1 and promoted aggressive behaviors of HCC (**Fig. 1Q**) 63. Similarly, H19 also promoted invasive behaviors through the miR-520a-3p/LIMK1 axis (**Fig. 1R**) 64.

H19 is also thought to be involved in chemotherapeutic resistance in HCC patients. Ding et al. 65 reported that silencing H19 reduced expression of the chemoresistance gene MDR1 by blocking MAPK/ERK signaling. Xu et al. 66 revealed that knocking-down H19 decreased miR-675 expression, which increased sorafenib sensitivity by inhibiting EMT in HCC cells. Together, these data revealed that H19 functioned as an oncogene in HCC.

#### H19 in gallbladder and bile duct tumors

Both gallbladder cancer (GBC) and cholangiocarcinoma (CCC) are low incidence tumors of the digestive system. Due to early metastasis, only a limited number of GBC or CCC cases can be resected, and the 5-year OS rate is only 20%-40% 67, 68. Therefore, understanding the pathogenesis of GBC and CCC is vital to reveal therapeutic targets.

Wang et al. 69 reported that knocking-down H19 decreased GBC cell growth, causing them to arrest in the G0/G1 phase *via* regulating miR-194-5p/AKT2 signaling (**Fig. 1S**). Another group discovered that H19 expression was higher in GBC tissues than in normal bladder tissues, and was positively correlated with tumor size and lymphatic metastasis. Increasing H19 expression promoted invasion and EMT by regulating Twist1 expression in GBC cells 70. Additionally, H19 functioned as a ceRNA of miR-342-3p to increase cell growth and invasion by enhancing FOXM1 expression in GBC cells (**Fig. 1T**) 71.

According to previous research, H19 promotes CCC cell migration and invasion by targeting CXCR4 and IL-6 *via* sponging miR-372/miR-373 and let-7a/b, respectively (**Fig. 1U and 1V**) 72. Additionally, silencing H19 promoted apoptosis and inhibited growth, migration, and invasion by reversing EMT in CCC cells 73. HIF1α-mediated H19 overexpression in CCC cells promoted proliferation, migration, and invasion *via* regulating the miR-612/Bcl-2 axis (**Fig. 1W**) 74. Overall, these data confirmed that H19 had an oncogenic activity in GBC and CCC and represented a promising diagnostic target.

#### H19 in pancreatic cancer

Pancreatic cancer is the least common digestive tumor, but is the fourth leading cause of death from cancer in the United States 43. Despite significant efforts of researchers to study the pathogenesis of pancreatic cancer, its five-year OS rate remains extremely low 75. Therefore, understanding the pathogenesis of pancreatic cancer is crucial for the development of successful treatments.

A recent study revealed that H19 expression was increased in pancreatic cancer compared with normal pancreatic tissue. Silencing H19 in pancreatic cancer cells led to decrease HMGA2 expression and blocked cell migration and invasion by regulating let-7 (**Fig. 1X**) 76. Another study showed that knocking-down H19 impaired the viability and growth of pancreatic cancer cells by decreasing E2F1 expression 77. Two years later, the same group demonstrated that H19 regulated E2F1 expression by sponging miR-675, which served as an underlying biomarker for diagnosing pancreatic cancer (**Fig. 1Y1**) 78. *In situ* hybridization rates of H19 also suggested it had an important role in pancreatic cancer metastasis, which implied that suppressing H19 could be a novel treatment for pancreatic cancer 79. Recently, it was reported that H19 promoted pancreatic cancer cell invasion and metastasis *via* increasing cell adhesion and cancer stem cell self-renewal by regulating CD24 and integrin expression 80. Another group found that knocking-down H19 inhibited the growth and migration of pancreatic cancer cells *via* altering the miR-194/PFTK1 signaling (**Fig. 1Z**) 81. Studies to better understand these related molecular mechanisms showed that increased H19 expression promoted chemoresistance, EMT, migration, and invasion through the miR-675-3p/SOCS5 axis (**Fig. 1Y2**) 82. Moreover, H19 increased the expression of VGF to activate the PI3K/AKT/CREB signaling pathway and promote aggressive phenotypes of pancreatic cancer 83. In summary, H19 played a vital role in the prognosis of pancreatic cancer.

### The role of H19 in respiratory system tumors

#### H19 in nasopharyngeal cancer (NPC)

NPC is a malignant tumor of the respiratory system, and most NPC patients are diagnosed in advanced stages 84. Because of its high sensitivity, radiotherapy is the primary treatment for NPC, but NPC often relapses after treatment 85. Therefore, discovering novel biomarkers and therapeutic strategies could be pivotal for NPC.

**Figure 2:**
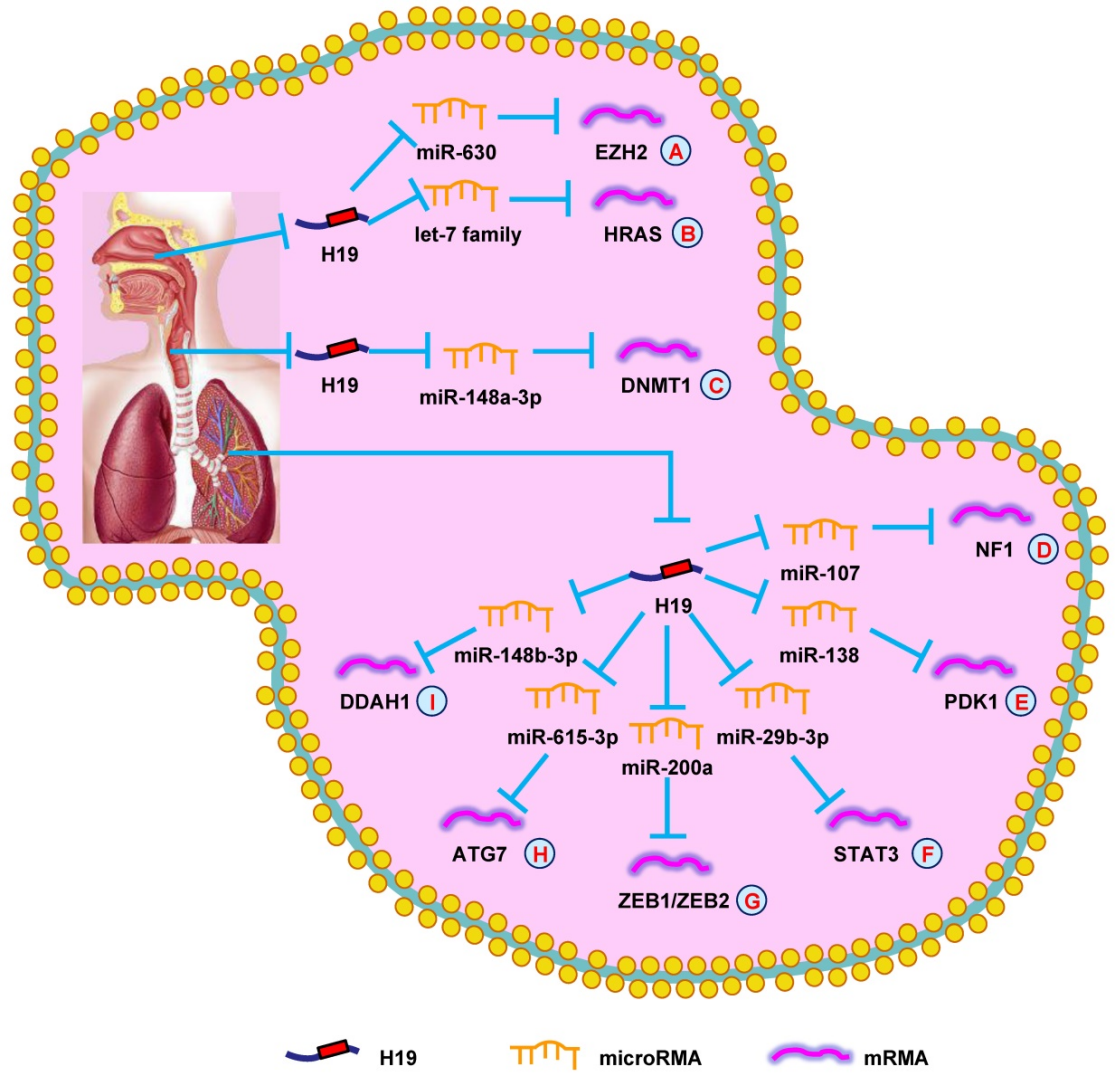
** H19 mediates mechanisms involved in respiratory system tumors.** (**A**). H19 could promote the expression of EZH2 by targeting miR-630. (**B**). H19 could promote the expression of HRAS by targeting let-7 family. (**C**). H19 could promote the expression of DNMT1 by targeting miR-148a-3p. (**D**). H19 could promote the expression of NF1 by targeting miR-107. (**E**). H19 could promote the expression of PDK1 by targeting miR-138. (**F**). H19 could promote the expression of STAT3 by targeting miR-29b-3p. (**G**). H19 could promote the expression of ZEB1/ZEB2 by targeting miR-200a. (**H**). H19 could promote the expression of ATG7 by targeting miR-615-3p. (**I**). H19 could promote the expression of DDAH1 by targeting miR-148b-3p.

In 2003, it was found that H19 was highly expressed in undifferentiated human NPC cell lines, but not in well-differentiated NPC cells. Additionally, it was demonstrated that hypomethylation of the CpG site in the H19 promoter region induced abnormal H19 expression in the well-differentiated NPC cells. Thus, hypermethylation of the H19 promoter region could be a significant epigenetic marker that played a vital role in the differentiation of NPC cells and the transcriptional silencing of imprinted genes 86. Li et al. 87 found that H19 suppressed E-cadherin expression and promoted NPC cell invasion by regulating the miR-630/EZH2 pathway, which suggested a possible therapy for NPC (**Fig. 2A**). In another study, increased H19 expression was associated with poorer prognosis. Mechanistically, H19 showed oncogenic activity through the let-7/HRAS pathway and promoted NPC oncogenesis and metastasis (**Fig. 2B**) 88. Additionally, upregulating H19 promoted the growth of NPC cells and decreased the chemosensitivity. Silencing H19 could be an effective method to suppress tumor growth 89. Together, these data supported the conclusion that H19 functioned as an oncogene and promoted NPC progression.

#### H19 in laryngeal cancer

Laryngeal cancer is a common tumor of the respiratory system, among which laryngeal squamous cell carcinoma (LSCC) is the main subtype. In 2021, an estimated 12,620 new cases will be diagnosed, and approximately 3,770 patients will die from this LSCC 43. Therefore, it is urgent to find new diagnostic biomarkers and novel therapies for LSCC.

Wu et al. 90 revealed that H19 expression was increased in LSCC. Silencing H19 in LSCC suppressed growth, migration, and invasion. H19 performed its biological activity in LSCC by targeting the miR-148a-3p/DNMT1 pathway (**Fig. 2C**). In summary, the data suggested that H19 played an important role in LSCC development and could be a therapeutic target.

#### H19 in lung cancer

Lung cancer is the malignancy with the highest mortality in the world. In 2021, approximately 235,760 patients will be diagnosed with lung and bronchial cancer in the United States, resulting in approximately 131,880 deaths 43. The pathogenesis of lung cancer is not well understood, although great progress has been achieved in recent decades. Therefore, sufficient research into lung cancer will help us defeat it.

Since 2015, many scientists have focused their attention on the role of H19 in lung cancer. Cui et al. 91 discovered that H19 expression was higher in non-small cell lung cancer (NSCLC) compared with normal lung tissues. H19 expression was induced by c-Myc and promoted mitotic progression *via* regulating miR-107 in NSCLC cells. A year later, another group revealed a similar phenomenon, in that knocking-down H19 suppressed the growth of NSCLC cells by regulating c-Myc transcription. H19 could be a novel therapeutic target and diagnostic marker for NSCLC 92. Additionally, the relationship between H19 and chemotherapeutic resistance was revealed in lung adenocarcinoma for the first time in 2017 93. Increased H19 expression was negatively correlated with cisplatin response in lung adenocarcinoma patients, which was associated with increased cell growth and metastasis and a cell-cycle arrest. Another study of NSCLC indicated that H19 functioned *via* exosomes in NSCLC cells. H19 was secreted into exosomes, mediated by hnRNPA2B1, and induced gefitinib resistance 94. Moreover, FOXF2 was found to promote the progression of NSCLC cells by mediating decreased PTEN expression through H19 95. Moreover, H19 was involved in methylation-mediated lung cancer progression. Finally, it was demonstrated that silencing H19 suppressed growth and EMT while promoting apoptosis of lung cancer cells through suppressing the CDH1 promoter 96.

Recently, several researcher groups have shown that H19 promotes lung cancer progression through lncRNA-miRNA-mRNA network. H19 promoted NSCLC progression by modulating NF1 expression *via* competitively binding to miR-107 (**Fig. 2D**) 97. Additionally, overexpressing H19 stimulated cell proliferation *via* the miR-138/PDK1 axis in NSCLC (**Fig. 2E**) 98. H19 also promoted EMT and cell viability by modulating the miR-29b-3p/STAT3 axis (**Fig. 2F**) 99. In another study, the H19/miR-200a/ZEB1/ZEB2 axis was shown to be involved in the growth and metastasis of lung cancer (**Fig. 2G**) 100.

Drug resistance is a major factor leading to chemotherapy failure in lung cancer patients. Pan et al. 101 validated that exosomal H19 expedited erlotinib resistance through the miR-615-3p/ATG7 axis, providing a new diagnostic and therapeutic target for NSCLC (**Fig. 2H**). Moreover, H19 facilitated resistance to gefitinib through the miR-148b-3p/DDAH1 axis in lung adenocarcinoma, offering a novel insight into resistance to EGFR inhibitors (**Fig. 2I**) 102. Together, these studies demonstrated that H19 participated in lung cancer progression and functioned as a diagnostic biomarker and therapeutic target.

### The role of H19 in breast cancer (BC)

BC is the greatest threat to women's health. It is estimated that in 2021, BC will account for 30% of new diagnoses and 15% of deaths of all cancers in the United States 43. Most BC cases occur in women >50-year-old; however, the incidence is rising in younger women. The prevalence of BC is 1.9% and 10.5% in women aged 20-34 and 35-44, respectively 103. Therefore, it is necessary to explore the etiological mechanisms of BC.

A recent study showed that H19 was induced by estrogen, and had higher expression in estrogen receptor (ER)-positive BC than in ER-negative BC. Moreover, increasing H19 expression accelerated BC cell growth, which could serve as a predictive factor for BC 104. Subsequently, multiple investigators have taken notice of the oncogenic role of H19; a study revealed that H19 expression was significantly correlated to ER, progesterone receptor, c-erbB-2, and lymph node metastasis in BC patients 105. Furthermore, H19 expression in postoperative plasma was lower than in samples taken before surgery, which could be an early prognostic monitoring factor for BC.

Additionally, H19 also plays an important role in BC drug resistance. Si et al. 106 indicated that H19, functioning as a downstream target of ERα, restrained apoptosis in response to paclitaxel treatment by suppressing transcription of NOXA and BIK. Silencing H19 restored paclitaxel chemosensitivity through the AKT pathway in BC cells 107. Furthermore, increasing H19 expression resulted in resistance to paclitaxel and anthracyclines. Silencing H19 increased drug sensitivity through the CUL4A-ABCB1/MDR1 pathway 108. Tamoxifen is another drug commonly used to treat ER+ BC patients. However, tamoxifen resistance resulted in recurrence and reduced OS in BC patients. A report showed that knocking-down H19 helped overcome tamoxifen and fulvestrant resistance by blocking c-MET and Notch signaling 109. Gao et al. 110 and Wang et al. 111 showed that silencing H19 elevated tamoxifen sensitivity by inhibiting cell growth or autophagy, which provided a novel option in fighting BC. Additionally, H19 knockdown restored trastuzumab sensitivity in BC 112 and restored doxorubicin (ADM) resistance by attenuating cell viability and colony-forming ability 113. Also, another recent study showed that blocking H19 decreased cell proliferation, migration, invasion, EMT, and induced a cell cycle arrest by targeting the p53/TNFAIP8 axis in triple-negative BC 114. Additionally, lncRNA PTCSC3 curbed cell growth by suppressing H19 in triple-negative BC 115.

**Figure 3:**
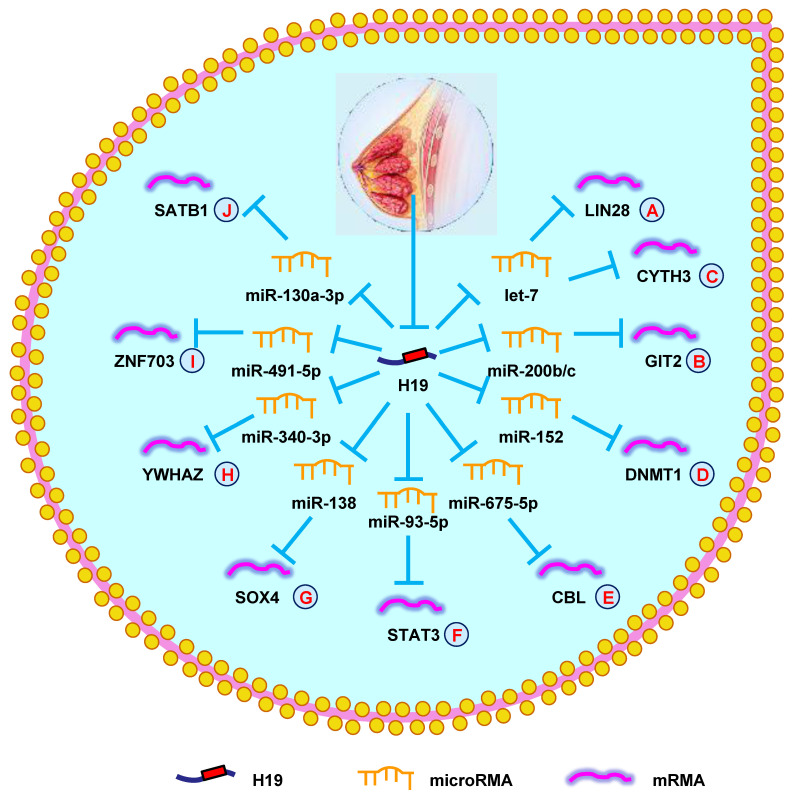
** H19 mediates mechanisms involved in breast cancer.** (**A**). H19 could promote the expression of LIN28 by targeting let-7. (**B**). H19 could promote the expression of GIT2 by targeting miR-200b/c. (**C**). H19 could promote the expression of CYTH3 by targeting let-7. (**D**). H19 could promote the expression of DNMT1 by targeting miR-152. (**E**). H19 could promote the expression of CBL by targeting miR-675-5p. (**F**). H19 could promote the expression of STAT3 by targeting miR-93-5p. (**G**). H19 could promote the expression of SOX4 by targeting miR-138. (**H**). H19 could promote the expression of YWHAZ by targeting miR-340-3p. (**I**). H19 could promote the expression of ZNF703 by targeting miR-491-5p. (**J**). H19 could promote the expression of SATB1 by targeting miR-130a-3p.

More recently, hundreds of investigators have further explored the lncRNA-miRNA-mRNA network in BC. In 2017, a study on BC showed that H19, let-7, and LIN 28 formed a double-negative feedback loop that played a vital role in BC formation. Further studies into the underlying mechanisms revealed that H19 functioned as a ceRNA of let-7, leading to increased LIN 28 expression (**Fig. 3A**) 116. Another report demonstrated a similar result that the H19/let-7/LIN 28 network also increased autophagy by suppressing EMT in BC 117. Moreover, H19 was found to mediate mesenchymal-to-epithelial transition and EMT *via* serving as a sponge for let-7 and miR-200b/c and regulating their targets Cyth3 and Git2 in BC (**Fig. 3B and 3C**) 118. Li et al. 119 showed that H19 enhanced cell growth and invasion through the miR-152/DNMT1 axis, providing a new mechanism for BC development (**Fig. 3D**). Another group found that Huaier extract decreased the viability of BC cells by inducing apoptosis through the H19/miR-675-5p/CBL axis (**Fig. 3E**) 120. Since 2019, myriad regulatory networks have been found to be involved in the occurrence and development of BC. The newly identified network H19/miR-93-5/STAT3 was shown to promote an aggressive phenotype of BC cells (**Fig. 3F**) 121. Silencing H19 suppressed invasive behaviors by modulating the miR-138/SOX4 axis in BC (**Fig. 3G**) 122. Yan et al. 123 uncovered that H19 functioned as a ceRNA to accelerate BC progression by regulating the miR-340-3p/YWHAZ axis, providing a potential therapeutic and prognostic biomarker for BC (**Fig. 3H**). Another report indicated that H19 increased growth, invasion, and migration in BC cells by sponging miR-491-5p to suppress ZNF703 (**Fig. 3I**) 124. Finally, silencing H19 inhibited BC tumorigenesis by regulating the miR-130a-3p/SATB1 axis (**Fig. 3J**) 125. These findings indicated that H19 was a novel oncogene that promoted BC progression.

### The role of H19 in genitourinary system tumors

#### H19 in endometrial cancer

Endometrial cancer is a rare malignancy of the female reproductive system. Although the incidence of endometrial cancer is not very high, it can cause significant pain to patients. Therefore, it is essential to clarify the underlying mechanisms of this malignancy.

**Figure 4:**
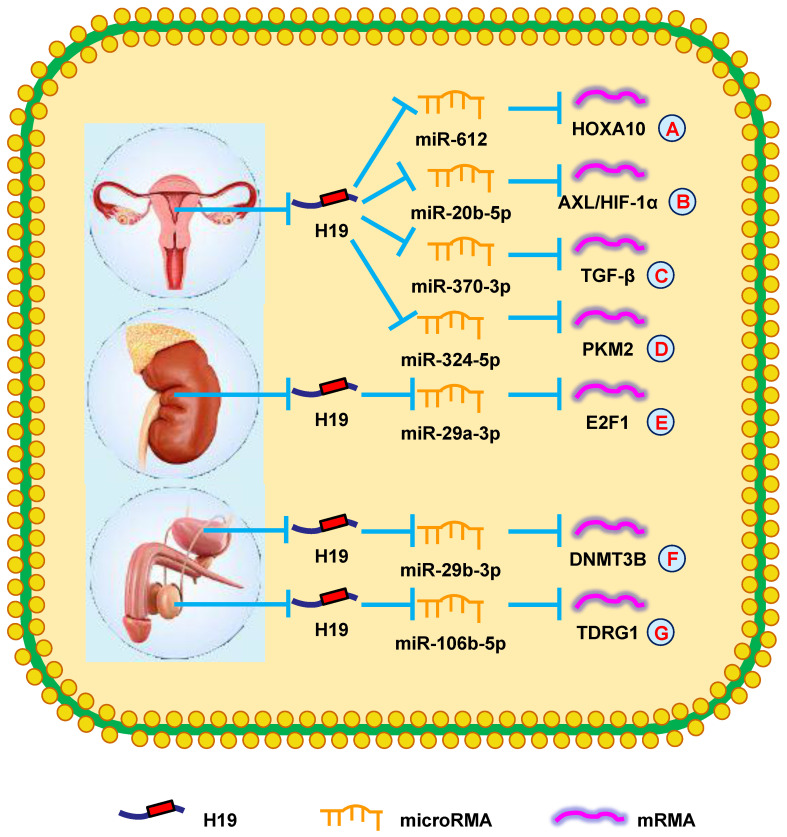
** H19 mediates mechanisms involved in genitourinary system tumors.** (**A**). H19 could promote the expression of HOXA10 by targeting miR-612. (**B**). H19 could promote the expression of AXL/HIF-1α by targeting miR-20b-5p. (**C**). H19 could promote the expression of TGF-β by targeting miR-370-3p. (**D**). H19 could promote the expression of PKM2 by targeting miR-324-5p. (**E**). H19 could promote the expression of E2F1 by targeting miR-29a-3p. (**F**). H19 could promote the expression of DNMT3B by targeting miR-29b-3p. (**G**). H19 could promote the expression of TDRG1 by targeting miR-106b-5p.

Zhang et al. 126 reported high H19 levels in endometrial cancer. Increased H19 levels promoted HOXA10 expression, which increased cell growth by targeting miR-612 (**Fig. 4A**). Another group showed that H19 induced the aggressive phenotype of endometrial cancer by targeting miR-20b-5p/AXL/HIF-1α signaling, providing a further target for treating endometrial cancer (**Fig. 4B**) 127. These findings indicated that H19 participated in endometrial cancer progression.

#### H19 in ovarian cancer (OC)

OC is an important tumor of the female genitourinary system. In 2021, OC is expected to account for 5% of all deaths of women from cancer in the United States 43. Therefore, there is an urgent need to uncover therapeutic targets in OC.

Silencing H19 was shown to inhibit cell proliferation by regulating certain cell cycle- and apoptosis-related proteins 128. Moreover, increasing H19 expression led to higher TGF-β levels, which promoted EMT of OC cells *via* antagonizing miR-370-3p, which suggested this pathway could be a potential therapeutic target (**Fig. 4C**) 129. Another report suggested that suppressing H19 with ginsenoside 20(S)-Rg3 increased the repression of PKM2 by miR-324-5p, thereby repressing OC tumorigenesis. Similarly, H19 mediates drug resistance in OC (**Fig. 4D**) 130. Sajadpoor et al. 131 showed that valproic acid inhibited H19 expression and blocked cell growth and cisplatin resistance *via* the EZH2/p21/PTEN pathway. In summary, these studies provided novel insights into the mechanisms of H19 in OC and could be developed into OC therapies.

#### H19 in renal cell carcinoma (RCC)

RCC is a common malignancy of the urinary system. Early-stage RCC is difficult to detect due to a lack of typical clinical manifestations. Therefore, investigating the molecular mechanisms of RCC is critical.

Wang et al. 132 showed that increased H19 expression was associated with poorer prognosis and advanced clinical stage in RCC patients. Knocking-down H19 in RCC cells attenuated their proliferation, invasion, and migration. Additionally, silencing H19 suppressed E2F1 expression by sponging miR-29a-3p and restrained cell migration and invasion (**Fig. 4E**) 133. These findings showed that H19 could be a therapeutic target in RCC.

#### H19 in bladder cancer

Bladder cancer is the most prevalent and fatal tumor of the urinary system 43. Because there are no obvious clinical manifestations in early stages, many bladder cancer patients are diagnosed in advanced stages. Therefore, novel therapies to fight bladder cancer are urgently needed.

Studies to better understand the specific molecular mechanisms of bladder cancer have revealed that increased H19 expression promoted bladder cancer cell metastasis by suppressing E-cadherin 134, 135. Furthermore, it was reported that increased H19 expression accelerated cell proliferation by modulating ID2 expression in bladder cancer 136. Recently, another study revealed that YAP1-enhanced H19 overexpression was associated with poorer clinicopathological prognoses of bladder cancer patients 137. Two years later, Lv et al. 138 showed that H19 attenuated the inhibitory effect of DNMT3B by functioning as a ceRNA for miR-29b-3p in bladder cancer (**Fig. 4F**). Additionally, Wang et al. 139 proposed that exosomal H19 expression was increased in bladder cancer patients and that these patients had reduced OS compared with other patients. In summary, H19 played a vital in bladder cancer prognosis and could be the target of novel bladder cancer treatments.

#### H19 in testicular tumors

Although testicular tumors account for a small percentage of malignancies in men, they are life-threatening to young men. Testicular neoplasms are difficult to detect in early stages because of their atypical clinical symptoms. Thus, it is essential to define more efficient diagnostic markers.

A new report revealed that H19 expression was elevated in cisplatin-resistant seminoma cells. H19 increased TDRG1 expression by sponging miR-106b-5p to stimulate cell survival in cisplatin-based chemotherapeutic conditions (**Fig. 4G**) 140. These results demonstrated that H19 could be a novel therapeutic target for chemoresistant testicular tumors.

### The role of H19 in nervous system tumors

Glioma is a common tumor of the nervous system. Due to its rapid progression and highly aggressive nature, the 5-year OS rate of glioma patients is only 15 months 141. Therefore, discovering the pathogenesis of glioma and novel therapeutic methods is important for glioma patients.

**Figure 5:**
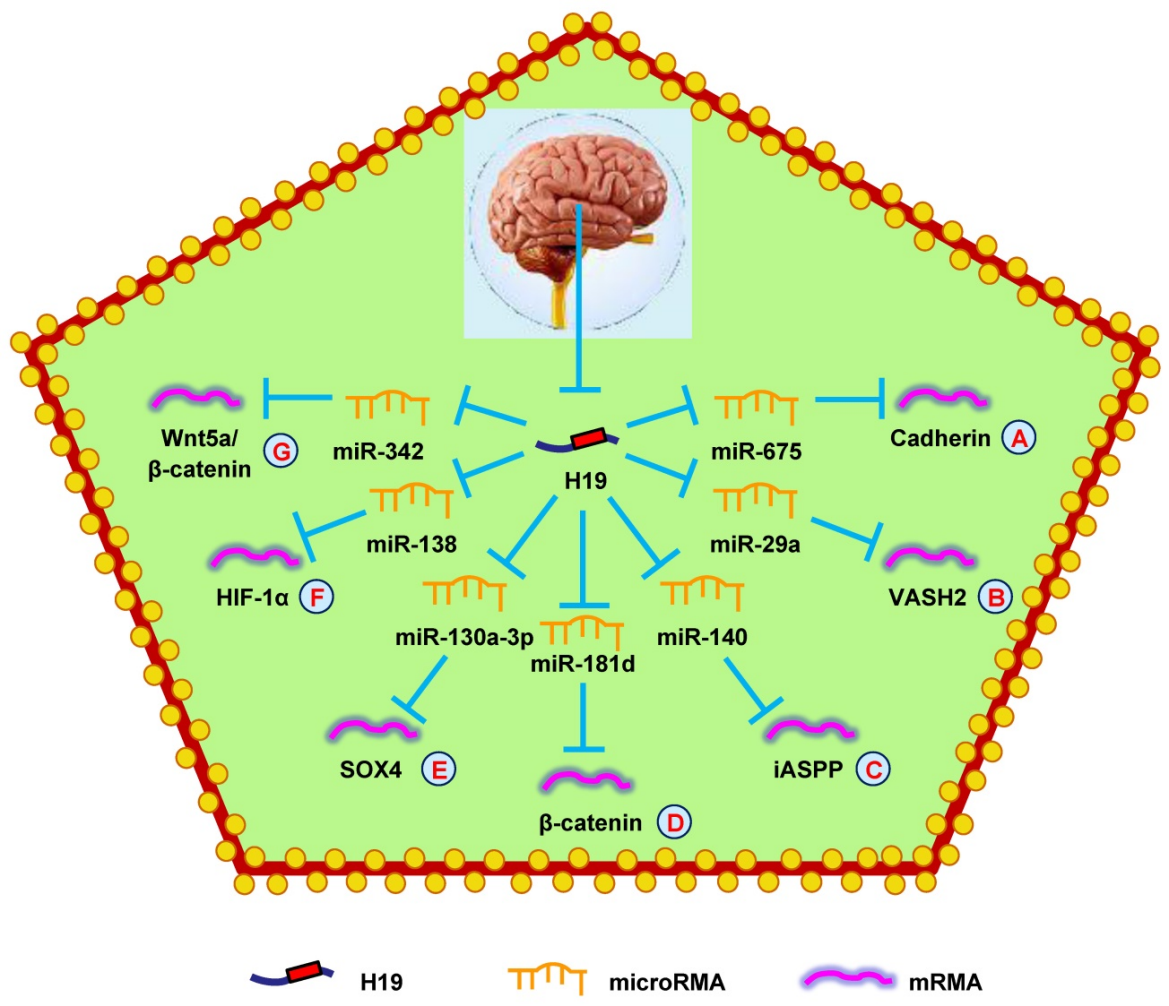
** H19 mediates mechanisms involved in nervous system tumors.** (**A**). H19 could promote the expression of Cadherin by targeting miR-675. (**B**). H19 could promote the expression of VASH2 by targeting miR-29a. (**C**). H19 could promote the expression of iASPP by targeting miR-140. (**D**). H19 could promote the expression of β-catenin by targeting miR-181d. (**E**). H19 could promote the expression of SOX4 by targeting miR-130a-3p. (**F**). H19 could promote the expression of HIF-1α by targeting miR-138. (**G**). H19 could promote the expression of Wnt5a/β-catenin by targeting miR-342.

A recent report showed that H19 was elevated in glioma cells and contributed to maintain the stemness properties and malignant behaviors of glioma cells 142. Another report showed that increased H19 expressed stimulated the tumorigenicity of glioma cells 143. Subsequently, H19 was confirmed to bind to EZH2 and regulate glioma cell viability, migration, and invasion by repressing NKD1 144. Additionally, knocking-down H19 suppressed the growth, migration, invasion, and cell cycle progression of glioma cells and increased apoptosis by attenuating Wnt/β-catenin signaling 145. In 2014, Shi et al. 146 first identified that miRNAs were involved in H19-mediated glioma progression. They reported that H19, mediated by miR-675, promoted cell proliferation in glioma. They found that H19 was upregulated in glioma cells and tissues, and was negatively correlated with patient survival (**Fig. 5A**). Another group came to a similar conclusion 147. Not long after, a study reported that H19 promoted cell growth and invasion by repressing miR-152 148.

H19 has also been found to regulate glioma progression through miRNA-mRNA network. Downregulation of H19 decreased VASH2 expression and inhibited tumor angiogenesis by upregulating miR-29a (**Fig. 5B**) 149. Another study showed that H19 regulated cell proliferation and metastasis by controlling miR-140-mediated iASPP expression in glioma, which could be a new therapeutic biomarker for glioma (**Fig. 5C**) 150. Another study found that hypoxia facilitated H19 expression, which relieved β-catenin suppression by binding miR-181d, enhancing the invasion and migration of glioma cells (**Fig. 5D**) 151. Moreover, H19 also competed with SOX4 by sponging miR-130a-3p to influence EMT, migration, and invasion in glioma (**Fig. 5E**) 152. Liu et al. 153 discovered that overexpressing H19 promoted the growth, invasion, and migration of glioma cells by serving as a ceRNA and regulating miR-138/HIF-1α signaling (**Fig. 5F**) 153. Additionally, a mechanistic study revealed that H19 accelerated cell proliferation and metastasis by modulating Wnt5a/β-catenin signaling* via* miR-342, providing novel therapeutic targets in glioma (**Fig. 5G**) 154.

H19 is also involved in glioma drug resistance. Jiang et al. 155 indicated significantly increased H19 levels in temozolomide (TMZ)-resistant glioma patients compared with TMZ-sensitive patients. H19 played a vital role in TMZ-resistance in glioma by altering the expression of drug resistance genes such as MRP, MDR, and ABCG2. Similarly, another study showed that H19 attenuated TMZ-resistance in glioma cells by inhibiting EMT *via* suppressing the Wnt/β-catenin pathway 156. Finally, H19 induced TMZ resistance in glioma cells *via* activating NF-κB 157. Together, these data demonstrated that H19 was an oncogene in glioma that could be a therapeutic target.

### The role of H19 in tumors of other systems

#### The role of H19 in lymphoma

Lymphoma is a class of malignant tumors that derive from the lymphatic hematopoietic system. Hodgkin's lymphoma (HL) is a major lymphoma subtype that most often occurs in people over 55-years-old and between 15- and 35-years-old. Therefore, it is meaningful to look for diagnostic biomarkers of HL.

Wang et al. 158 reported that H19 was upregulated in HL tissues and inversely associated with OS in HL patients. H19 stimulated HL cell proliferation through AKT. These data confirmed that H19 promoted HL progression and functioned as an oncogene.

#### The role of H19 in myeloma

Myeloma is a malignant hematological tumor characterized by excessive proliferation of bone marrow plasma cells. Although there has been recent progress in treatments for myeloma, these are associated with adverse reactions such as severe infection, myelosuppression, neutropenia, and drug resistance. Therefore, improving myeloma treatment, including new drugs or combination therapies, is an urgent issue.

**Figure 6:**
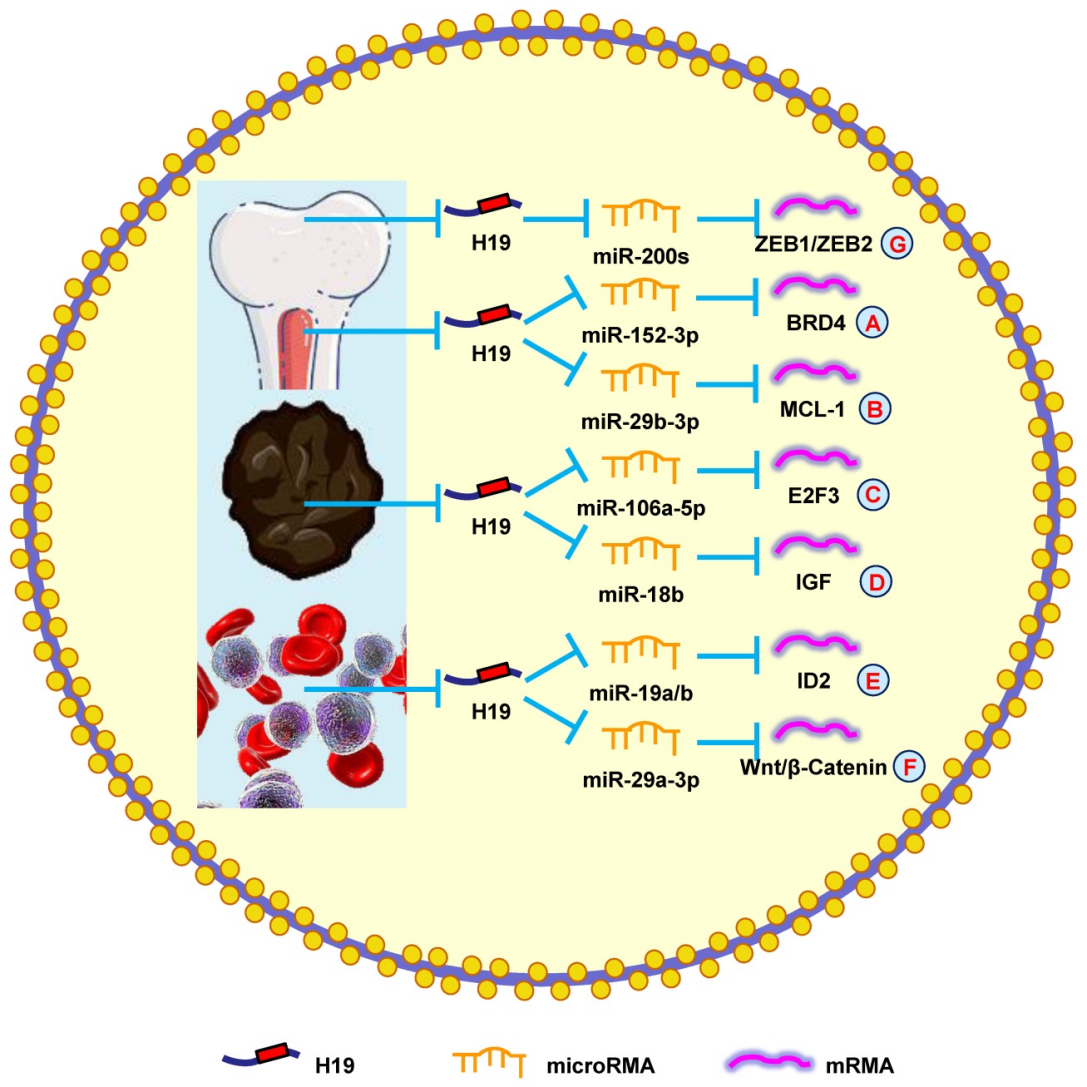
** H19 mediates mechanisms involved in tumors of other systems.** (**A**). H19 could promote the expression of BRD4 by targeting miR-152-3p. (**B**). H19 could promote the expression of MCL-1 by targeting miR-29b-3p. (**C**). H19 could promote the expression of E2F3 by targeting miR-106a-5p. (**D**). H19 could promote the expression of IGF by targeting miR-18b. (**E**). H19 could promote the expression of ID2 by targeting miR-19a/b. (**F**). H19 could promote the expression of Wnt/β-Catenin by targeting miR-29a-3p. (**G**). H19 could promote the expression of ZEB1/ZEB2 by targeting miR-200s.

A previous study showed that H19 was elevated in myeloma patients and cell lines. The severity of myeloma was also shown to be associated with H19 levels in the serum of patients, suggesting that H19 could also be a therapeutic target 159. Another study reported that blocking H19 along with reducing NF-κB expression blocked the growth of myeloma cells 160. Silencing H19 attenuated the tumorigenesis of myeloma cells by blocking BRD4 expression through the miR-152-3p pathway (**Fig. 6A**) 161.

Moreover, H19 is involved in myeloma drug resistance. Pan et al. 8 revealed that H19 reduced chemosensitivity to bortezomib *via* upregulating MCL-1 by functioning as a miRNA sponge and sequestering miR-29b-3p (**Fig. 6B**). Additionally, upregulating H19 and AKT suppressed apoptosis, while silencing H19 and AKT accelerated apoptosis. Anti-H19 was a potential way to block drug resistance in myeloma 162. These results confirmed that H19 was a therapeutic target in myeloma.

#### The role of H19 in melanoma

The World Health Organization estimates that 66,000 people die from skin cancer worldwide each year; melanoma is responsible for 80% of those deaths 163. Therefore, understanding the pathogenesis of melanoma is vitally important for exploring new therapeutic targets.

It was previously reported that H19 accelerates the growth of melanoma cells by functioning as a miR-106a-5p sponge and increasing E2F3 expression (**Fig. 6C**) 9. H19 is associated with poor prognoses, which means it could be a novel therapeutic target for melanoma. Zhu et al. 164 found that overexpressing H19 promoted the growth and invasion of melanoma cells by upregulating MMP2 and MMP9. Another study discovered that silencing H19 suppressed melanoma cell migration and invasion by deactivating NF‑κB expression *via* the PI3K/Akt pathway 165. Furthermore, H19 overexpression in melanoma patients was correlated with poor clinical prognosis, such as lymph node metastasis, distant metastasis, and shorter OS. Silencing H19 inhibited the migration and invasion of melanoma cells and induced melanoma cell apoptosis after a G0/G1 arrest 166. Finally, H19 promoted cisplatin-resistance by regulating miR-18b/IGF signaling, which suggests it could be a therapeutic target for melanoma (**Fig. 6D**) 22. Overall, these data indicated that H19 had an oncogenic role in melanoma and represented a novel therapeutic target.

#### The role of H19 in leukemia

Leukemia is a malignant tumor with high morbidity and mortality in both men and women 43. Recently, several groups have tried to explore the underlying mechanisms of leukemia, but there has not been a breakthrough. Hence, a full understanding of leukemia development would facilitate better disease management.

According to the study by Zhao et al. 167, silencing H19 decreased ID2 expression by competitive binding to miR-19a and miR-19b, which restrained cell growth (**Fig. 6E**). Expectedly, ectopic H19 expression was associated with shorter OS and lower complete remission, which was confirmed using the Gene Ontology Omnibus and The Cancer Genome Atlas datasets. It was also determined that H19 produced oncogenic effects through the downstream gene ID2 in leukemia 168. Finally, H19 was shown to sequester miR-29a-3p to promote cell growth and inhibit apoptosis in leukemia through the Wnt/β-catenin pathway (**Fig. 6F**) 169. Taken together, these results suggested that H19 played an oncogenic role in leukemia.

#### The role of H19 in osteosarcoma

Osteosarcoma is a rare malignant tumor that occurs in both adolescents and children. The highest incidence of osteosarcoma is between the ages of 10 and 20. With improved surgical techniques and chemotherapy regimens, the 5-year OS rate has increased from 20% to 70% 170. However, it remains urgent to explore effective diagnostic and prognostic biomarkers for osteosarcoma to further improve OS.

Li et al. 171 reported that H19 increased metastasis *via* elevating ZEB1 and ZEB2 expression by combining with the miR-200 family (**Fig. 6G**). Another group revealed that patients with high H19 expression had a shorter OS compared with those with low H19 expression. Downregulating H19 attenuated cell invasion and migration by suppressing NF-κB signaling 172. These findings indicated that H19 was a potential biomarker that could be used to diagnose and treat osteosarcoma.

## Conclusion and future perspectives

Cancer has been the leading cause of death in China since 2010, becoming a dominant public health issue in the country and worldwide 173. Because early clinical symptoms are often not obvious and there is a lack of effective biological markers, many patients are diagnosed in advanced stages. The current lack of efficient therapeutic strategies for advanced tumors directly contributes to the high mortality rate for many malignancies. Therefore, studies to identify early-stage diagnostic markers and therapeutic targets have been performed by many researchers.

LncRNAs can function as oncogenes or tumor suppressor genes, and thus are involved in the occurrence and development of many different tumor types. Several lncRNAs are potential diagnostic and/or prognostic biomarkers including lncRNA HOTAIR 174, lncRNA MALAT1 175, lncRNA MEG3 176, lncRNA PVT1 177, lncRNA XIST 178, and H19 27. Although H19 is one of the most studied lncRNAs, many molecular mechanisms remain unelucidated. Therefore, a comprehensive study of its downstream effectors and upstream regulatory mechanisms may provide a novel perspective to better counteract H19 in cancer.

In this review, we highlighted several examples of increased H19 expression and its role in cancer development, progression, prognosis, and treatment. H19 functions through a variety of mechanisms, such as interactions with miRNAs and/or target proteins to maintain cancer characteristics. Abnormal overexpression of H19 has been identified to be tightly associated with clinicopathological characteristics of the different cancers. H19 can also competitively combine with mRNAs by antagonizing miRNAs, revealing a regulatory network model of “H19-miRNAs-mRNAs.” Additionally, H19 overexpression is an important reason for chemotherapy resistance in malignant tumors.

Although further investigations are needed to expand our understanding of the molecular function of H19 in a more comprehensive way, the clinical application of H19 has aroused great interest. Recent studies have shown that H19 can be released from a variety of cancers and can be detected in patients' serum, which could be used for early detection and establishment of personalized treatments. For example, plasma H19 levels have been proposed by some scholars as a predictive biomarker for GC, BC, and lung cancer, and as an important tool for monitoring cancer development 32, 105, 179. In addition, Sorin et al. 180 found that liver metastatic growth in treated animals was significantly reduced by using a plasmid approach to selectively kill H19-expressing cells with the diphtheria toxin A chain gene controlled by the H19 promoter (DTA-H19/BC-819). The method of BC-819 instillations to limit tumor recurrence is conducted in phase 1/2a trial for ovarian cancer and in phase 2b trial for BC 181, 182. Finally, H19 increases resistance to ADM, 5-Fu, PTX, gefitinib, TMZ, sorafenib, tamoxifen, and MTX in almost all types of cancer, indicating the importance of designing anti-H19 therapy to improve the response of cancer patients to a broad range of treatment regimens.

Overall, several lines of evidence indicated that H19 plays important roles in tumor development and progression. H19 expression is proposed as a novel biomarker for many tumors. Antineoplastic drugs targeting H19 could be used to more accurately and safely treat malignant tumors.

## Figures and Tables

**Table 1:** Functional characterization of H19 in digestive system tumors.

Tumor types	Expression	Role	Function role	miRNAs	Related genes	References
Esophageal cancer	upregulation	oncogene	proliferation and metastasis	let-7c	STAT3/EZH2/Catenin	28
Esophageal cancer	upregulation	oncogene	proliferation, migration, and stemness	miR-22-3p	WNT1	29
Gastric cancer	upregulation	oncogene	proliferation, migration, invasion, and metastasis	miR-675	CALN1	20
Gastric cancer	upregulation	oncogene	proliferation	miR-675	RUNX1	37
Gastric cancer	upregulation	oncogene	proliferation and invasion	miR-675	RUNX1	38
Gastric cancer	upregulation	oncogene	proliferation and apoptosis	miR-675	FADD	39
Gastric cancer	upregulation	oncogene	proliferation and invasion	miR-141	ZEB1	40
Gastric cancer	upregulation	oncogene	/	let-7c	HER2	41
Gastric cancer	upregulation	oncogene	proliferation, invasion, migration, and EMT	miR-22-3p	Snail1	42
Colorectal cancer	upregulation	oncogene	proliferation	miR-675	RB	44
Colorectal cancer	upregulation	oncogene	EMT	miR-138/200a	ZEB1/ZEB2	45
Colorectal cancer	upregulation	oncogene	migration and invasion	/	RAS-MAPK	48
Colorectal cancer	upregulation	oncogene	migration, invasion, and EMT	/	hnRNPA2B1/Raf-1/ERK	50
Colorectal cancer	upregulation	oncogene	proliferation	miR-200a	β-Catenin	51
Colorectal cancer	upregulation	oncogene	motility, EMT, invasion, and migration	miR-29b-3p	PGRN	52
Colorectal cancer	upregulation	oncogene	invasion, migration, and EMT	miR-194-5p	FoxM1	53
Colorectal cancer	upregulation	oncogene	EMT and metastasis	miR-22-3p	MMP14	54
Colorectal cancer	upregulation	oncogene	migration and invasion	miR-138	HMGA1	55
Colorectal cancer	upregulation	oncogene	apoptosis	miR-141	β-Catenin	56
Colorectal cancer	upregulation	oncogene	proliferation and migration	miR-675-5p	VDR	57
Colorectal cancer	upregulation	oncogene	autophagy	miR-194-5p	SIRT1	11
Hepatocellular cancer	upregulation	oncogene	proliferation, migration, invasion, and EMT	miR-22	/	60
Hepatocellular cancer	upregulation	oncogene	proliferation, migration, and invasion	miR-326	TWIST1	61
Hepatocellular cancer	upregulation	oncogene	proliferation, migration, invasion, and apoptosis	miR-15b	CDC42/PAK1	62
Hepatocellular cancer	upregulation	oncogene	migration and invasion	miR-193b	MAPK1	63
Hepatocellular cancer	upregulation	oncogene	proliferation, metastasis, and apoptosis	miR-520a-3p	LIMK1	64
Gallbladder cancer	upregulation	oncogene	proliferation and invasion	miR-194-5p	AKT2	69
Gallbladder cancer	upregulation	oncogene	invasion and proliferation	miR-342-3p	FOXM1	71
Cholangiocarcinoma	upregulation	oncogene	migration and invasion	let-7a/b	IL-6	72
Cholangiocarcinoma	upregulation	oncogene	migration and invasion	miR-372/373	CXCR4	73
Cholangiocarcinoma	upregulation	oncogene	proliferation, migration, and invasion	miR-612	BCL-2	74
Pancreatic cancer	upregulation	oncogene	migration, invasion, and EMT	let-7	HMGA2	76
Pancreatic cancer	upregulation	oncogene	proliferation and apoptosis	/	E2F1	77
Pancreatic cancer	upregulation	oncogene	proliferation	miR-675	E2F1	78
Pancreatic cancer	upregulation	oncogene	proliferation and invasion	miR-194	PFTK1	81
Pancreatic cancer	upregulation	oncogene	migration, invasion, and EMT	miR-675-3p	SOCS5/STAT3	82
Pancreatic cancer	upregulation	oncogene	proliferation, migration, and invasion	/	VGF/PI3K/AKT/CREB	83

**Table 2:** Main characteristics of the studies included in the review of digestive system tumors

Study	Tumor types	Sample size (Normal: Tumor)	Detection Method	P value (p value)	TNM (p value)	LNM (p value)	DM (p value)	OS (p value)	DFS (p value)	References
Huang	Esophageal cancer	(133 : 133)	qRT-PCR	p<0.05	p=0.001	/	p=0.000	/	/	26
Tan	Esophageal cancer	(64 : 64)	qRT-PCR	p<0.01	p=0.01	p=0.007	/	/	/	27
Chen	Esophageal cancer	(30 : 30)	qRT-PCR	p<0.05	/	/	/	/	/	28
Zhang	Gastric cancer	(80 : 80)	qRT-PCR	p<0.001	p=0.016	p=0.002	/	p=0.007	/	31
Zhou	Gastric cancer	(70 : 70)	qRT-PCR	p<0.0001	/	/	/	/	/	32
Chen	Gastric cancer	(128 : 128)	qRT-PCR	p<0.01	p=0.005	/	p=0.041	p<0.001	/	33
Hashad	Gastric cancer	(32 : 30)	qRT-PCR	p<0.001	p=0.014	/	/	/	/	34
Jia	Gastric cancer	(284 : 284)	qRT-PCR	p<0.05	p=0.026	p<0.001	/	p=0.001	/	21
Li	Gastric cancer	(74 : 74)	qRT-PCR	p=0.017	/	p=0.027	p=0.001	p=0.036	/	20
Zhou	Gastric cancer	(15 : 15)	qRT-PCR	p<0.05	/	/	/	/	/	40
Wei	Gastric cancer	(24 : 24)	qRT-PCR	p<0.001	/	p=0.015	/	/	/	41
Gan	Gastric cancer	(40 : 40)	qRT-PCR	p<0.05	p<0.05	p<0.05	/	/	/	42
Tsang	Colorectal cancer	(40 : 40)	qRT-PCR	p=0.001	/	/	/	/	/	44
Liang	Colorectal cancer	(30 : 30)	qRT-PCR	p<0.05	/	/	/	/	/	45
Han	Colorectal cancer	(83 : 83)	qRT-PCR	p<0.01	p=0.008	/	/	p=0.002	p=0.029	46
Chen	Colorectal cancer	(96 : 96)	qRT-PCR	p<0.01	p=0.046	/	/	/	p<0.01	47
Zhang	Colorectal cancer	(60 : 60)	qRT-PCR	p<0.001	/	p=0.010	p=0.042	p=0.013	/	50
Yang	Colorectal cancer	(30 : 30)	qRT-PCR	p<0.01	/	/	/	/	/	51
Ding	Colorectal cancer	(185 : 185)	qRT-PCR	p<0.05	p=0.033	p=0.002	/	p<0.001	/	53
Li	Colorectal cancer	(214 : 214)	qRT-PCR	p<0.05	p=0.011	p<0.001	/	p<0.001	/	54
Ren	Colorectal cancer	(10 : 10)	qRT-PCR	p=0.0169	/	/	/	/	/	56
Li	Hepatocellular cancer	(36 : 36)	qRT-PCR	p<0.01	p=0.044	p=0.018	p=0.007	/	/	60
Zhou	Hepatocellular cancer	(46 : 46)	qRT-PCR	p<0.01	/	/	/	/	/	62
Ding	Hepatocellular cancer	(42 : 42)	qRT-PCR	p<0.01	/	/	/	/	/	65
Wang	Gallbladder cancer	(20 : 20)	qRT-PCR	p<0.05	/	/	/	/	/	69
Wang	Gallbladder cancer	(24 : 24)	qRT-PCR	p<0.05	/	p=0.017	/	/	/	70
Wang	Gallbladder cancer	(36 : 36)	qRT-PCR	p<0.05	/	/	/	/	/	71
Xu	Cholangiocarcinoma	(56 : 56)	qRT-PCR	p<0.001	p=0.0145	/	/	p=0.0007	/	73
Ma	Pancreatic cancer	(20 : 20)	qRT-PCR	p<0.05	/	/	/	/	/	76
Ma	Pancreatic cancer	(30 : 30)	qRT-PCR	p=0.007	/	/	/	/	/	77
Sun	Pancreatic cancer	(45 : 45)	qRT-PCR	p<0.01	p<0.001	/	p<0.001	p=0.024	/	81
Ji	Pancreatic cancer	(39 : 39)	qRT-PCR	p<0.01	p<0.001	p=0.044	p=0.001	/	p<0.001	83

**Table 3:** Functional characterization of H19 in respiratory, genitourinary, and nervous system tumors.

Tumor types	Expression	Role	Function role	miRNAs	Related genes	References
Nasopharyngeal cancer	upregulation	oncogene	invasion	miR-630	EZH2	87
Nasopharyngeal cancer	upregulation	oncogene	proliferation, migration, and invasion	let-7 family	HRAS	88
Laryngeal cancer	upregulation	oncogene	proliferation, migration, and invasion	miR-148a-3p	DNMT1	90
Lung cancer	upregulation	oncogene	cell cycle	miR-107	/	91
Lung cancer	upregulation	oncogene	migration, invasion, and EMT	/	CDH1	96
Lung cancer	upregulation	oncogene	proliferation and migration	miR-107	NF1	97
Lung cancer	upregulation	oncogene	proliferation	miR-138	PDK1	98
Lung cancer	upregulation	oncogene	viability, proliferation, and apoptosis	miR-29b-3p	STAT3	99
Lung cancer	upregulation	oncogene	proliferation, migration, and invasion	miR-200a	ZEB1/ZEB2	100
Lung cancer	upregulation	oncogene	proliferation, migration, and invasion	miR-615-3p	ATG7	101
Lung cancer	upregulation	oncogene	proliferation, migration, and invasion	miR-148b-3p	DDAH1	102
Endometrial cancer	upregulation	oncogene	viability	miR-612	HOXA10	126
Endometrial cancer	upregulation	oncogene	proliferation, migration, EMT, and apoptosis	miR-20b-5p	AXL/HIF-1α	127
Ovarian cancer	upregulation	oncogene	EMT	miR-370-3p	TGF-β	129
Ovarian cancer	upregulation	oncogene	Warburg effect	miR-324-5p	PKM2	130
Renal cancer	upregulation	oncogene	migration and invasion	miR-29a-3p	E2F1	133
Bladder cancer	upregulation	oncogene	migration	/	EZH2	134
Bladder cancer	upregulation	oncogene	proliferation	/	ID2	136
Bladder cancer	upregulation	oncogene	proliferation, migration, invasion, mobility, and EMT	miR-29b-3p	DNMT3B	138
Seminoma	upregulation	oncogene	chemotherapeutic sensitivity	miR-106b-5p	TDRG1	140
Glioma	upregulation	oncogene	viability, migration, and invasion	/	NKD1	144
Glioma	upregulation	oncogene	proliferation, migration, invasion, cell cycle, and apoptosis	/	Wnt/β-Catenin	145
Glioma	upregulation	oncogene	invasion	miR-675	Cadherin	146
Glioma	upregulation	oncogene	proliferation	miR-675	/	147
Glioma	upregulation	oncogene	proliferation and invasion	miR-152	/	148
Glioma	upregulation	oncogene	proliferation, migration, and tube formation	miR-29a	VASH2	149
Glioma	upregulation	oncogene	proliferation	miR-140	iASPP	150
Glioma	upregulation	oncogene	migration and invasion	miR-181d	β-catenin	151
Glioma	upregulation	oncogene	migration, invasion, and EMT	miR-130a-3p	SOX4	152
Glioma	upregulation	oncogene	proliferation, migration, invasion, and angiogenesis	miR-138	HIF-1α	153
Glioma	upregulation	oncogene	proliferation, migration, and angiogenesis	miR-342	Wnt5a/β-catenin	154

**Table 4:** Main characteristics of the studies included in the review of respiratory, genitourinary, and nervous system tumors.

Study	Tumor types	Sample size (Normal: Tumor)	Detection Method	P value (p value)	TNM (p value)	LNM (p value)	DM (p value)	OS (p value)	DFS (p value)	References
Li	Nasopharyngeal cancer	(30 : 31)	qRT-PCR	p<0.05	/	/	/	/	/	87
Zhang	Nasopharyngeal cancer	(17 : 48)	qRT-PCR	p<0.01	/	/	/	p=0.0195	/	88
Wu	Laryngeal cancer	(82 : 82)	qRT-PCR	p<0.01	p<0.01	p<0.01	/	p=0.003	/	90
Cui	Lung cancer	(30 : 30)	qRT-PCR	p<0.01	/	/	/	/	/	91
Zhang	Lung cancer	(70 : 70)	qRT-PCR	p<0.001	p<0.001	/	/	/	/	92
Xu	Lung cancer	(48 : 48)	qRT-PCR	p<0.001	/	/	/	p=0.0125	/	95
Gao	Lung cancer	(60 : 60)	qRT-PCR	p<0.05	/	/	/	/	/	96
Qian	Lung cancer	(36 : 36)	qRT-PC	p<0.05	/	/	/	/	/	97
Huang	Lung cancer	(20 : 20)	qRT-PCR	p<0.001	/	/	/	/	/	98
Liu	Lung cancer	(305 : 305)	qRT-PCR	p<0.05	p=0.026	/	/	p=0.001	/	99
Zhao	Lung cancer	(22 : 22)	qRT-PCR	p<0.001	/	/	/	/	/	100
Zhang	Endometrial cancer	(43 : 43)	qRT-PCR	p<0.001	/	/	/	p=0.0489	/	126
Zhu	Endometrial cancer	(36 : 36)	qRT-PCR	p<0.001	/	/	/	/	/	127
Zhu	Ovarian cancer	(70 : 70)	qRT-PCR	p<0.01	/	/	/	/	/	128
Wang	Renal cancer	(92 : 92)	qRT-PCR	p<0.05	p=0.023	p=0.013	p=0.002	p<0.05	/	132
He	Renal cancer	(30 : 30)	qRT-PCR	p<0.01	/	/	/	/	/	133
Luo	Bladder cancer	(41 : 41)	qRT-PCR	p=0.0034	/	/	/	/	/	134
Zhu	Bladder cancer	(48 : 48)	qRT-PCR	p<0.01	/	p<0.01	p<0.01	/	/	135
Luo	Bladder cancer	(24 : 24)	qRT-PCR	p=0.0015	/	/	/	/	/	136
Li	Bladder cancer	(19 : 40)	qRT-PCR	p<0.05	/	/	/	/	/	137
Lv	Bladder cancer	(35 : 35)	qRT-PCR	p<0.001	p=0.0154	p=0.0456	/	/	/	138
Wang	Bladder cancer	(52 : 52)	qRT-PCR	p<0.001	p=0.009	/	/	p=0.002	/	139
Jiang	Glioma	(30 : 30)	qRT-PCR	p<0.0001	/	/	/	/	/	143
Guan	Glioma	(60 : 60)	qRT-PCR	p<0.05	/	/	/	p<0.05	/	145
Zhang	Glioma	(35 : 35)	qRT-PCR	p<0.001	/	/	/	p<0.005	/	147
Zhao	Glioma	(28 : 28)	qRT-PCR	p<0.01	/	/	/	/	/	150
Wu	Glioma	(15 : 22)	qRT-PCR	p=0.0003	/	/	/	/	/	151
Zhou	Glioma	(30 : 30)	qRT-PCR	p<0.001	/	/	/	/	/	154

**Table 5:** Functional characterization of H19 in breast cancer.

Tumor types	Expression	Role	Function role	miRNAs	Related genes	References
Breast cancer	upregulation	oncogene	proliferation, migration, invasion, and cell cycle	/	TNFAIP8	114
Breast cancer	upregulation	oncogene	clonogenicity, migration, and mammosphere-forming ability	let-7	LIN28	116
Breast cancer	upregulation	oncogene	autophagy and EMT	let-7	LIN28	117
Breast cancer	upregulation	oncogene	EMT	miR-200b/c and let 7b	GIT2 and CYTH3	118
Breast cancer	upregulation	oncogene	proliferation and invasion	miR-152	DNMT1	119
Breast cancer	upregulation	oncogene	viability	miR-675-5p	CBL	120
Breast cancer	upregulation	oncogene	migration and invasion	miR-93-5p	STAT3	121
Breast cancer	upregulation	oncogene	proliferation, migration, invasion, cell cycle, EMT, and apoptosis	miR-138	SOX4	122
Breast cancer	upregulation	oncogene	proliferation, metastasis, invasion, EMT, and apoptosis	miR-340-3p	YWHAZ	123
Breast cancer	upregulation	oncogene	proliferation, migration, invasion, and apoptosis	miR-491-5p	ZNF703	124
Breast cancer	upregulation	oncogene	proliferation, migration, invasion, and apoptosis	miR-130a-3p	SATB1	125

**Table 6:** Main characteristics of the studies included in the review of breast cancer.

Study	Tumor types	Sample size (Normal: Tumor)	Detection Method	P value (p value)	TNM (p value)	LNM (p value)	DM (p value)	OS (p value)	DFS (p value)	References
Zhang	Breast cancer	(24 : 24)	qRT-PCR	p=0.018	/	/	/	/	/	105
Li	Breast cancer	(60 : 60)	qRT-PCR	p<0.001	/	/	/	/	/	114
Wang	Breast cancer	(69 : 69)	qRT-PCR	p<0.05	/	/	/	/	/	115
Peng	Breast cancer	(20 : 20)	qRT-PCR	p<0.01	/	/	/	p=0.004	/	116
Zhou	Breast cancer	(48 : 48)	qRT-PCR	p<0.05	/	/	/	/	/	118
Li	Breast cancer	(45 : 45)	qRT-PCR	p<0.05	/	/	/	/	/	119
Si	Breast cancer	(40 : 40)	qRT-PCR	p<0.01	/	/	/	p<0.05	/	122
Wang	Breast cancer	(20 : 20)	qRT-PC	p<0.05	/	/	/	/	/	124
Zhong	Breast cancer	(50 : 50)	qRT-PCR	p<0.01	/	/	/	/	/	125

**Table 7:** Functional characterization of H19 in other system tumors.

Tumor types	Expression	Role	Function role	miRNAs	Related genes	References
Lymphoma	upregulation	oncogene	proliferation	/	AKT	158
Myeloma	upregulation	oncogene	proliferation and apoptosis	miR-152-3p	BRD4	161
Myeloma	upregulation	oncogene	proliferation, migration, viability, cell cycle, and apoptosis	miR-29b-3p	MCL-1	8
Myeloma	upregulation	oncogene	chemotherapeutic sensitivity	/	AKT	162
Melanoma	upregulation	oncogene	glucose metabolism and growth	miR-106a-5p	E2F3	9
Melanoma	upregulation	oncogene	proliferation, migration, and invasion	/	NF‑κB	165
Melanoma	upregulation	oncogene	chemotherapeutic sensitivity	miR-18b	IGF	22
Leukemia	upregulation	oncogene	proliferation	miR-19a/b	ID2	167
Leukemia	upregulation	oncogene	proliferation and apoptosis	/	ID2	168
Leukemia	upregulation	oncogene	proliferation and apoptosis	miR-29a-3p	Wnt/β-Catenin	169
Osteosarcoma	/	oncogene	migration and invasion	miR-200s	ZEB1/ZEB2	171
Osteosarcoma	upregulation	oncogene	migration and invasion	/	NF‑κB	172

**Table 8:** Main characteristics of the studies included in the review of other system tumors.

Study	Tumor types	Sample size (Normal: Tumor)	Detection Method	P value (p value)	TNM (p value)	LNM (p value)	DM (p value)	OS (p value)	DFS (p value)	References
Wang	Lymphoma	(60 : 40)	qRT-PCR	p<0.001	/	/	/	p=0.0269	/	158
Pan	Myeloma	(80 : 67)	qRT-PCR	p<0.0001	/	/	/	/	/	159
Zheng	Myeloma	(30 : 30)	qRT-PCR	p<0.01	/	/	/	/	/	161
Wang	Myeloma	(50 : 60)	qRT-PCR	p<0.0001	/	/	/	/	/	162
Luan	Melanoma	(30 : 30)	qRT-PCR	p<0.0001	p<0.001	/	/	p<0.05	/	9
Liao	Melanoma	(49 : 49)	qRT-PCR	p<0.05	/	/	/	/	/	165
Shi	Melanoma	(82 : 82)	qRT-PCR	p<0.01	p=0.0076	p=0.0481	p=0.0153	p=0.021	/	166
An	Melanoma	(30 : 30)	qRT-PC	p<0.01	/	/	/	p=0.012	/	20
Zhao	Leukemia	(53 : 46)	qRT-PCR	p<0.001	/	/	/	/	/	167
Zhang	Leukemia	(36 : 161)	qRT-PCR	p=0.003	/	/	/	p=0.02	/	168
Zhao	Leukemia	(40 : 40)	qRT-PCR	p<0.01	/	/	/	/	/	169
Liao	Osteosarcoma	(40 : 40)	qRT-PCR	p<0.05	/	/	p=0.01	p=0.00322	/	172

## Abbreviations

lncRNAs: Long non-coding RNAs
H19: LncRNA H19
ncRNAs: Non-coding RNAs
EMT: Epithelial-mesenchymal transition
OS: Overall survival
miRNA: MicroRNA
EC: Esophageal cancer
ESCC: Esophageal squamous cell carcinoma
GC: Gastric cancer
CRC: Colorectal cancer
MTX: Methotrexate
5-Fu: 5-fluorouracil
HCC: Hepatocellular carcinoma
GBC: Gallbladder cancer
CCC: Cholangiocarcinoma
NPC: Nasopharyngeal cancer
LSCC: Laryngeal squamous cell carcinoma
NSCLC: Non-small cell lung cancer
BC: Breast cancer
ER: Estrogen receptor
PTX: Paclitaxel
ADM: Doxorubicin
OC: Ovarian cancer
RCC: Renal cell carcinoma
TMZ: Temozolomide
HL: Hodgkin's lymphoma
DTA: Diphtheria toxin A
